# Cohort profile and representativeness of participants in the Danish monozygotic twin study on migraine

**DOI:** 10.1007/s10654-025-01329-3

**Published:** 2025-11-03

**Authors:** Isa Amalie Olofsson, Jes Olesen, Kaare Christensen, Eva R. Hoffmann, Thomas Folkmann Hansen

**Affiliations:** 1 Department of Cellular and Molecular Medicine, Faculty of Health and Medical Sciences, University of Copenhagen, Copenhagen, Denmark; 2 Danish Headache Center, Department of Neurology, Rigshospitalet, Copenhagen University Hospital, Glostrup, Denmark; 3 The Danish Twin Registry, Department of Public Health, University of Southern Denmark, Odense, Denmark; 4 Department of Clinical Genetics, Odense University Hospital, Odense, Denmark; 5 Department of Clinical Biochemistry and Pharmacology, Odense University Hospital, Odense, Denmark; 6 Neurogenomic Group, Translational Research Centre, Rigshospitalet, Copenhagen University Hospital, Glostrup, Denmark

**Keywords:** Epidemiology, Genetics, Headache, Twin study, Co-twin control, Neurology

## Abstract

The Danish Monozygotic Twin Study on Migraine is a population-based twin study established in 2023–2024. The cohort was created to lay the foundation for innovative studies of the role of environmental, genetic, and epigenetic factors and their complex interactions in the pathogenesis of migraine. The aim of this paper is to describe data collection, content, characteristics of participants and to assess the representativeness of the cohort by comparing participants to non-responders. Danish monozygotic twins born between 1967 and 2000 were invited to participate. Self-reported questionnaires were sent out to 9,036 possible participants. The questionnaires assessed migraine and migraine subtypes, life satisfaction, resilience, stress, childhood trauma and the relationship of the participant to their family. Through linkage to the nationwide Danish registries the cohort contains individual level information on education, income, patient data from hospitals, prescription medication and childbirth. The Danish registries also enable longitudinal data collection on health outcomes. Individuals who responded to the migraine questionnaire were defined as participants. The cohort consists of 3,893 individuals, including 1,822 complete twin pairs, 1,173 individuals with migraine and 280 migraine discordant twin pairs. 123 participants were included in a substudy with a migraine diagnostic interview and collection of blood samples for both genetic and epigenetic studies. Comparison between participants and non-responders showed a higher participation rate among women. For both genders participants were older, had a higher level of education and a higher level of income compared to non-responders. Sociodemographic differences in participation should be considered to avoid biased estimates in future studies based on the cohort.

## Introduction

Migraine is a chronic complex neurovascular disorder that affects over 1 billion people worldwide [1]. Migraine is the 2nd leading cause of years lost to disability and the first among young women [2–4]. Despite the high prevalence and large burden of migraine, our understanding of the mechanisms behind migraine is still limited.

Population-based family and twin studies have estimated that genetic and environmental factors play an equal role in the development of migraine [5]. While both genetic and environmental risk factors have been extensive studied, their role in migraine etiology remains poorly understood. Genetic studies have identified multiple common genetic variants, but they only explain 11% of the total migraine phenotype [6]. Observational studies on the effects of lifestyle factors, socioeconomic factors and environmental pollution on the development of migraine, are often inconsistent [7–13]. So far, primarily childhood adverse events such as maltreatment or sexual abuse, have consistently been associated with migraine in several smaller studies [14–18]. Twin studies with monozygotic twins (MZ) offer a unique opportunity to account for the influences of genetic and shared environmental factors on migraine susceptibility, and could help identify modifiable risk factors and genetic biomarkers for migraine [19]. In the co-twin control design one twin in the pair is used as a matched control for the other twin [20]. Few studies have utilized discordant and concordant MZ twin pairs to understand the pathogenesis of migraine [12, 21–29].

To create a cohort with MZ twins discordant and concordant for migraine, it is necessary to have a large population to sample from. The Danish Twin Registry was initiated in 1954 with twins born since 1870, and is among the oldest twin registries in the world [30–32]. Today the Danish Twin Registry comprises more than 175,000 twins with the youngest individuals born in 2009 [32]. With the resources in the Danish Twin Registry it is possible to recruit participants for twin studies nationwide [32].

The Danish Monozygotic Twin Study on Migraine is a population-based twin study established in 2023–2024. The cohort is designed to lay the foundation for innovative studies of the role of environmental, genetic, and epigenetic factors and their complex interactions in the pathogenesis of migraine. Further, the cohort will be a source for studies of biomarkers associated with migraine. The aim of this paper is to describe data collection, content, characteristics of participants and to assess the representativeness of the study population by comparing participants to non-responders.

## Materials and methods

### Study population and recruitment

The Danish Monozygotic Twin Study on Migraine included Danish MZ twins born between 1967 and 2000. Possible participants were identified from the Danish Twin Registry [32]. The Danish Twin Registry is a nationwide cohort of twins, who have volunteered to participate in research. In the Danish Twin Registry zygosity for same-sex twin pairs was primarily assessed by questionnaire, but when available based on serological or genetic markers [30, 32]. The Danish Twin Registry questionnaire used for zygosity had an estimated 95% accuracy [33]. Individuals were eligible for inclusion in the cohort if they meet the following criteria:

- Registered in the Danish Twin Registry.
- Born between 1967 and 2000.
- Both individuals in a twin pair alive in April 2023.
- Classified as MZ based on questionnaire or serological/genetic markers.

From the Danish Twin Registry we identified 9,036 MZ twins (4,518 complete twin pairs) born between 1967 and 2000. In May 2023 possible participants received an invitation to the cohort by secure electronic mail. A reminder was sent by secure electronic mail to all non-responders in November 2023. Inclusion of participants concluded in June 2024.

### Data collection

#### Questionnaires

Participants were asked to complete a questionnaire comprising several validated instruments: A 33-item migraine questionnaire [34], a 5-item Satisfaction with Life Scale [35], an 18-item Adult Sibling Relationship Questionnaire – Very Short Form [36], a 3-item UCLA loneliness scale [37], as well as the first 17 items from the Resilience Scale for Adults [38] and 6 items on stress including traumatic childhood events.

The migraine questionnaire has been validated with a positive predictive value of 95% for all types of migraine combined [34]. The questionnaire was based on the International Classification of Headache Disorders, 3rd edition (ICHD-3) [39]. The questionnaire assessed migraine diagnosis, headache frequency and duration, pain characteristics, accompanying symptoms and aura symptoms. The questionnaire identified the migraine sub-types: migraine without aura, migraine with aura, menstrual migraine and high frequency migraine (≥ 8 migraine days per month).

#### Nationwide registries and longitudinal data collection

A personal identification number (CPR number) is given to all persons living in Denmark [40], which allows research data to be linked to all Danish nationwide registries. This is performed at Statistics Denmark (dst.dk), where datasets from the registries are stored at secure servers. Access to the deidentified individual-level data is given through a secure network connection. Through the Danish nationwide registries, it is possible to prospective follow participants and non-responders. Datasets from the Danish Medical Birth Registry, the Danish National Patient Registry, the Danish National Prescription Registry, the Population Education Register and the Income Statistics Register together with data from the Danish Civil Registration System were available for the cohort. The Danish Civil Registration System was established in 1968 for administrative purpose and contains information on all persons alive and with permanent residence in Denmark in 1968 or later [41]. The Danish Medical Birth Registry contains information from both mother and child related to pregnancy and childbirth [42]. The Danish Medical Birth Registry has existed since 1973, but several new variables have been added over the years. The Danish National Patient Registry contains individual-level data from all Danish hospitals since 1977 [43]. The Danish National Prescription Registry contains information of individual-level data on prescriptions filled at pharmacies since 1995 [44]. The Population Education Register contains information on the highest completed education authorized by the Danish Ministry of Education [45]. In 2008 the Population Education Register contained information from 96.4% of the Danish population aged 15–69 years [45]. The Income Statistics Register has been available since 1970 and is based on final tax assessments [46]. The Income Statistics Register includes all individuals who has submitted a tax return in Denmark [46].

The Danish registries enable nationwide longitudinal data collection on clinical and health outcomes based on diagnose codes registered at Danish hospitals or based on the prescription of any medicinal product filled at Danish pharmacies.

### Substudy of migraine discordant twin pairs

Based on the initial questionnaire responses, we identified twin pairs discordant for migraine. From November 2023 to January 2024 discordant twin pairs were invited to participate in a substudy by secure electronic mail. Participation included a diagnostic semi-structured clinical migraine interview and collection of two peripheral whole blood samples. Participants in the substudy provided informed consent to whole-genome sequencing and epigenetic analysis based on the collected blood samples.

#### Clinical interview

The clinical interview used a validated semi-structured interview based on the ICHD-3 criteria for migraine [39, 47, 48]. The interview was conducted face-to-face, by video consultation or by telephone. All interviews were conducted by the same medical doctor trained in headache diagnosis (I.A.O) and supervised by a senior headache expert (J.O). Participants were asked to describe headache without the use of any headache medication. Headache diagnoses were based on pain characteristics, accompanying symptoms and aura symptoms. The presence of menstrual-related migraine was recorded for women. Participants were asked about age of migraine onset and number of migraine attacks. Response to acute headache medication was documented. Somatic and psychiatric comorbidities were recorded together with lifestyle factors (height, weight, smoking status, pack years, alcohol consumption and physical activity).

#### Biological sample collection

Two standard peripheral blood samples (a total of 15 ml) were collected at the substudy visit. Visit sites were spread across Denmark (*n* = 8). Blood samples were collected with standard procedures for collection of peripheral blood samples by the same investigator (I.A.O). One blood sample was aliquoted into three cryotubes. All samples were stored in a -20 °C freezer.

### Data management

All data collected at baseline and from the substudy were recorded in the secure web-based electronic data management system, REDCap (Research Electronic Data Capture) [49]. Data from REDCap were securely transferred to Statistics Denmark to enable linkage with the nationwide registries based on the unique personal identifier. Data was deidentified by Statistics Denmark and stored at dedicated secure server accessible only by authorized researchers through an encrypted and logged network connection.

### Variables

Information of migraine was based on the self-reported migraine questionnaires. All sociodemographic characteristics of participants and non-responders were based on the nationwide registries. Gender and age were retrieved from the Danish Civil Registration System [41]. Age was used both as a continuous variable and categorized in four groups as: ≤29 years, 30–39 years, 40–49 years, ≥ 50 years. Information on the highest attained education was obtained from the Population Education Register, and categorized in five levels as: Primary education, Upper secondary education, Vocational education, Bachelors programmes, and Masters and PhD programmes according to the International Standard Classification of Education (ISCED) 2011 [45, 50]. Information on income was obtained from the Income Statistics Register and defined as yearly individual income in Danish kroner (DKK) [46]. Income was assessed both as a continuous variable and divided into quintiles. Quintiles were rounded to nearest 10,000 DKK: <110,000, 110,000 - <320,000, 320,000 - <430,000, 430,000 - <550,000 and ≥ 550,000 DKK/year.

### Statistical analyses

Individuals who responded to the migraine questionnaire were defined as participants. Individuals who did not respond to the migraine questionnaire were defined as non-responders. Descriptive characteristics were presented as number (percent) or median (interquartile range). Casewise concordance rate of migraine for complete twin pairs were calculates as:

$$\begin{aligned} & casewise\,concordance\,rate \\ & \quad =\frac{{2 \times number\,of\,concordant\,pairs~}}{{2 \times number\,of\,concordant\,pairs+number\,of\,discordant\,pairs}} \\ \end{aligned} $$

Comparison between participants and non-responders was done for gender, age, level of education and level of income. Binary logistic regression analyses were used to estimate odds ratios (OR) and corresponding 95% confidence intervals (CI) of participation in the cohort. As both gender and age differed significantly between participants and non-responders, the logistic regression analysis for gender was adjusted for age. We performed logistic regression analysis as a gender-specific analysis with men and women analyzed separate. The gender-specific logistic regression analysis estimated participation for age, level of education and level of income for each gender separate. The analyses for level of education and level of income were adjusted for age. No correction for multiple testing was performed. All analyses were performed with the statistical software R (version 4.4.1) and R-studio (version 2023.12.1).

## Results

### Study population

**Fig. 1:**
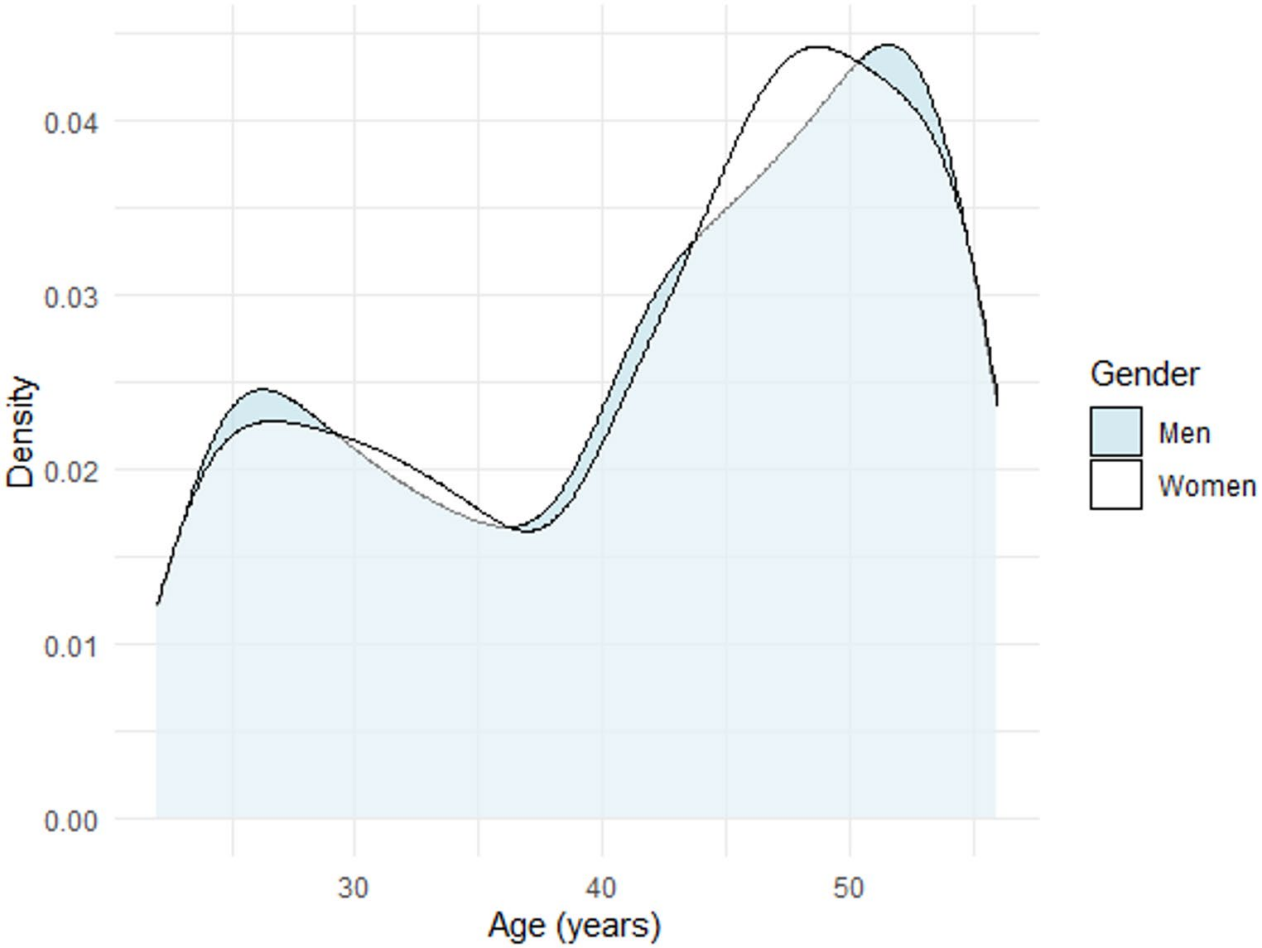
Density plot of age distribution of participants by gender

A total of 9,036 MZ twins (4,518 complete twin pairs) were contacted through secure electronic mail and invited to participate in the study. Of these, 3,893 individuals (43%) participated, including 1,822 complete twin pairs. There were more women (*n* = 2,420, 62%) than men among participants. The median age of participants was 44 years (interquartile range 33–51 years), see Table 1. There was a bimodal age distribution with fewer participants in the age of 30–40 years, see Fig. 1. There was no difference in age between men and women. In the cohort 78% (*n* = 3,044) of the participants consented to further contact with follow-up or future studies on migraine.

### Migraine

Based on the self-reported migraine questionnaire 1,173 (30%) participants fulfilled the ICHD-3 criteria for migraine. Of these, 524 (45%) participants with migraine fulfilled the diagnostic criteria for migraine without aura and 649 (55%) participants with migraine fulfilled the criteria for migraine with aura or for both migraine with aura and migraine without aura. For participants with migraine with aura most experienced visual aura (88%, *n* = 570) followed by sensory aura (31%, *n* = 200). High frequency migraine was reported in 39 participants. There was a higher prevalence of migraine in women (38%, *n* = 919) compared to men (17%, *n* = 252). Menstrual-related migraine was reported in 171 (19%) women with migraine.

The cohort included 280 migraine discordant twin pairs and 237 migraine concordant twin pairs. The case-wise concordance rate for migraine in the cohort was 0.63.

### Substudy of migraine discordant twin pairs

**Fig. 2:**
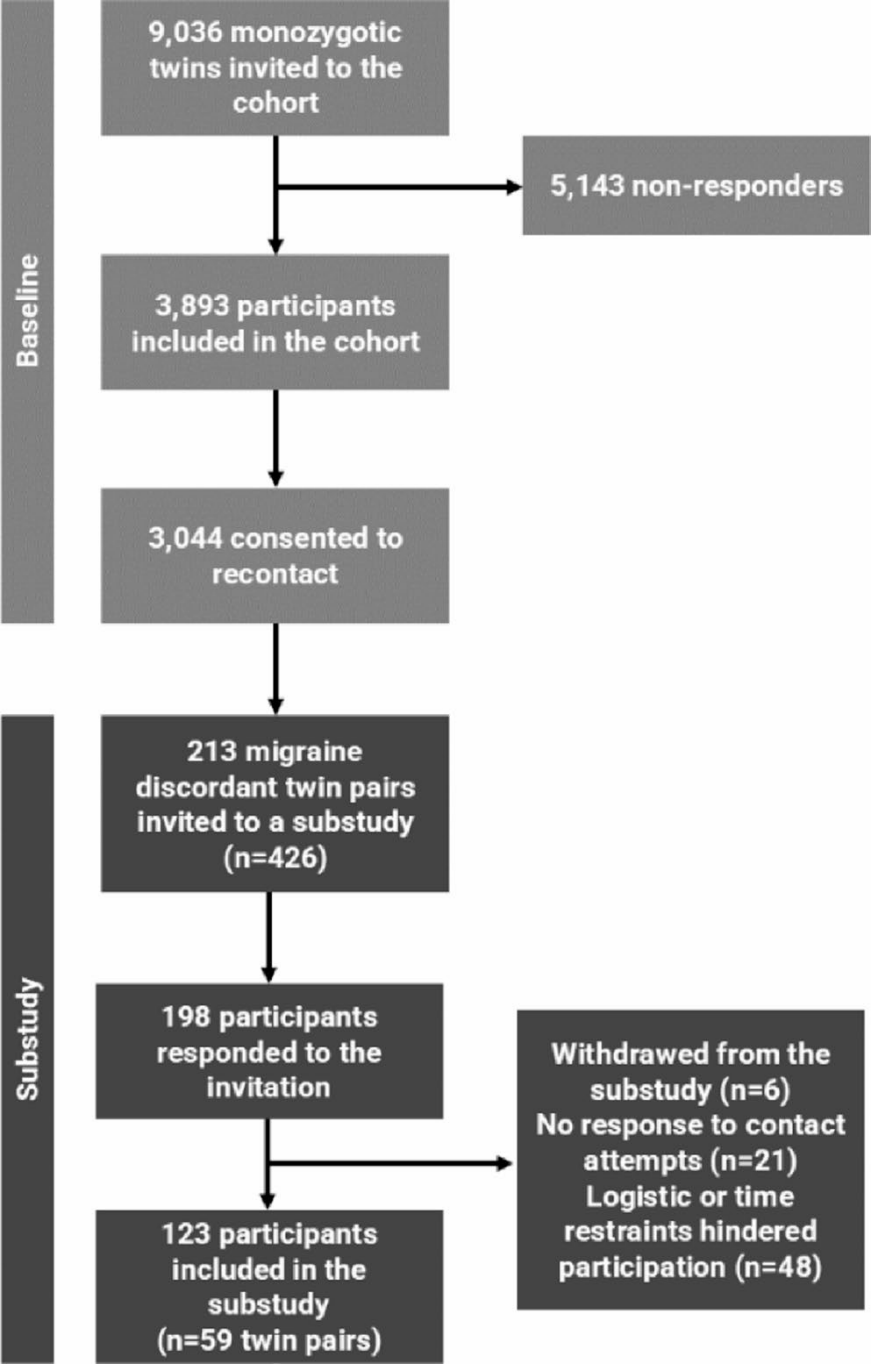
Flowchart of inclusion of participants at baseline and the substudy

A total of 213 migraine discordant twin pairs (*n* = 426 individuals) were invited to participate in a substudy. Of the invited participants, 198 (46%) participants responded, and interview and blood samples were collected from 123 participants, including 59 complete twin pairs. A flowchart of inclusion of participants at baseline and the substudy is shown in Fig. 2.

### Comparison of participants to non-responders

Of the 9,036 individuals invited to the study, 5,143 individuals were defined as non-responders. Demographic description of participants and non-responders are shown in Table 1.

**Table 1:** Demographic description of participants and non-responders

	Participants (*n* = 3,893)	Non-responders (*n* = 5,143)
Women	2,420 (62%)	2,224 (43%)
Age (median, IQR)	44 years (33–51 years)	41 years (30–48 years)
Income (median, IQR)	400,000 DKK/year (240,000–530,000 DKK/year)	360,000 DKK/year (130,000–500,000 DKK/year)
Education		
Primary education	252 (6%)	694 (14%)
Upper secondary education	307 (8%)	495 (10%)
Vocational education	1,120 (29%)	1,777 (35%)
Bachelors programmes	1,349 (35%)	1,377 (27%)
Masters and PhD programmes	864 (22%)	786 (15%)

IQR: Interquartile range. DKK: Danish kroner

Participants differed from non-responders based on both gender, age and socioeconomic determinants. Odds ratio and confidence interval for participation in the cohort is shown in Table 2. Women were more likely to participate then men (OR = 2.12, 95%CI 2.09–2.15). Participation rate increased with increasing age. The highest participation rate was seen in individuals aged > 49 years for both men and women (women: OR = 1.74, 95%CI 1.47–2.08, men: OR = 2.10, 95%CI 1.75–2.54), compared to the youngest age group of < 30 years. For both men and women participation rate increased with a higher level of education. The highest participation was seen in individuals with an education level of a masters or PhD programmes (women: OR = 2.26, 95%CI 2.18–2.35, men: OR = 3.51, 95%CI 3.37–3.65). For men, those with the highest individual income (≥ 550,000 DKK/year) were more likely to participate (OR = 2.48, 95%CI 2.40–2.56), compared to those with the lowest individual income (< 100,000 DKK/year). For women, those with a high-medium individual income of 430,000 - <550,000 DKK/year were more likely to participate (OR = 2.34, 95%CI 2.27–2.40), compared to those with the lowest individual income.

**Table 2:** Odds ratio and 95% confidence interval for participation in the cohort

	Women	Men
**Age**		
≤ 29 years	Reference	Reference
30–39 years	1.13 (0.94–1.36)	1.06 (0.87–1.29)
40–49 years	1.33 (1.13–1.56)	1.37 (1.15–1.64)
≥ 50 years	1.75 (1.47–2.08)	2.10 (1.75–2.54)
**Income**		
< 110,000 DKK/year	Reference	Reference
110,000 - <320,000 DKK/year	1.15 (1.12–1.18)	1.45 (1.40–1.51)
320,000 - <430,000 DKK/year	1.50 (1.46–1.54)	1.52 (1.47–1.58)
430,000 - <550,000 DKK/year	2.34 (2.27–2.40)	1.99 (1.93–2.06)
≥ 550,000 DKK/year	1.79 (1.73–1.85)	2.48 (2.40–2.56)
**Education**		
Primary education	Reference	Reference
Upper secondary education	1.44 (1.37–1.51)	1.67 (1.59–1.76)
Vocational education	1.56 (1.51–1.62)	1.87 (1.80–1.94)
Bachelors programmes	2.13 (2.06–2.21)	2.93 (2.82–3.05)
Masters and PhD programmes	2.26 (2.18–2.35)	3.51 (3.37–3.65)

DKK: Danish kroner. Income and education adjusted for age.

## Discussion

The Danish Monozygotic Twin Study on Migraine includes nationwide MZ twins born between 1967 and 2000. The cohort contains information on migraine and migraine subtypes, self-reported questionnaires on life satisfaction, resilience, stress, childhood trauma and the relationship to their family. Through linkage to the nationwide Danish registries the cohort contains individual-level information on education, income, patient data from hospitals, prescription medication and childbirth. Further, the Danish registries enable longitudinal data collection on health outcomes based on diagnosis codes at hospitals and based on the prescription of any medicinal product filled at pharmacies. The substudy included 123 twins with a migraine diagnostic interview and collection of blood samples for both genetic and epigenetic studies. The different sources of data included in the cohort make it possible to conduct a wide range of studies with twin designs.

Migraine diagnosis in participants was based on a validated self-reported questionnaire following ICHD-3 criteria for migraine and several migraine subtypes. The life-time prevalence of migraine in the cohort was 30% for all participants, 38% for women and 17% for men. This is higher than the corresponding life-time migraine prevalence of 25% for all (32% for women and 17% for men) in the general Danish population of a comparable age group [51]. It could be speculated that participants with migraine or with family members with migraine were more prone to participate in the study.

Participants in the cohort had a median age of 44 years with a bimodal age distribution. The drop in participation rates in 30–40 year olds have been reported in other population-based cohorts, and could be due to family formation [52, 53].

The comparison between participants and non-responders revealed significant differences in both the general characteristics and the socioeconomic variables. Participants were more likely to be women compared to men, and for both genders of greater age, with a higher level of education and a higher level of income compared to non-responders. This is in accordance with findings from other studies [54–56]. The difference in participation introduces a potential selection bias, that may affect both the internal and external validity of the cohort. Studies with a selected cohort can still be valid and generalizable, but future studies need to consider a possible selection bias [57]. Differences in age, income and level of education could affect associations with migraine based on an uneven distribution of stress between participants and non-responders. Studies indicate that higher levels of stress are associated with migraine susceptibility [15, 58, 59]. If the cohort has a selection bias with lower levels of stress in participants, it might introduce a collider bias. To mitigate a possible selection bias, directed acyclic graphs (DAGs) would make underlying assumptions transparent. Further statistical methods like inverse probability weighting and multiple imputation together with sensitivity analysis should be considered, depending on the research question.

The substudy included possible migraine discordant twin pairs for genetic and epigenetic analyses. In genetic studies a large sample size is often needed for adequate statistical power. When a co-twin control design is used for genetic studies, a much smaller sample size is required for a statistical power comparable to conventional case-control studies. For epigenetic studies on DNA methylation, with a heritability of migraine at 40–50% and a twin correlation of methylation of 0.3, a sample size of 50 migraine discordant MZ twin pairs would give a power >0.8, depending on the degree of environmental effect on DNA methylation [60].

### Strength and limitations

The Danish Monozygotic Twin Study on Migraine was designed with the purpose to examine the environmental, genetic, and epigenetic contributions to the pathogenesis of migraine. The study has several strengths: Participants were recruited nationwide, and the study included data on several variables. Twinning for MZ twins is a random event and twins are in general representative of the population they are drawn from [61]. A twin pregnancy carries increased risk of prenatal mortality and congenital abnormalities, and twins can further differ from singletons due to lower birth weight and gestational age [62]. However, for a large range of traits, diseases, behaviors and lifestyle parameters twins do not differ from singletons [61, 62]. For studies on migraine, the prevalence and clinical characteristics of migraine in twins have been compared to that in singletons, without any significant difference in both a Danish and a Swedish twin sample [63, 64]. For the diagnosis of migraine, a validated migraine questionnaire was used, reducing the risk of misclassification. Another strength is the linkage to the nationwide registries, with nearly complete follow-up data for many variables and a low risk of loss to follow-up. The study had an acceptable participation rate of 43% [65]. There are also some inherent limitations in the study: As participants were identified from the voluntary based Danish twin registry, there could be a further selection bias compared to the general population [31, 32]. The study only included MZ twins, and therefore has a limited sample size that needs to be considered when analytic methods are chosen. For the questionnaire data there is a risk of recall bias and misclassification of migraine cases. Migraine cases or incident migraine events can be identified based on hospital diagnosis codes and on the prescription of triptans from the nationwide Danish registries. However, International Classification of Diseases diagnose codes for migraine registered at hospitals are estimated to only capture 8% of the Danish migraine prevalence [66]. Anatomical Therapeutic Chemical (ATC) classification for triptans can be used as surrogate markers for migraine, but are estimated to only capture 12% of the Danish migraine prevalence [67].

### Data availability and collaboration

Data may be available on request if approved by the project committee and depending on ethical approval. For potential collaborations and secondary use of the data, the corresponding author can be contacted.

## Conclusion

The Danish Monozygotic Twin Study on Migraine is a population-based twin study established in 2023–2024. The cohort was created to lay the foundation for innovative studies of the role of environmental, genetic, and epigenetic factors and their complex interactions in the pathogenesis of migraine. Further, the cohort can be a source for studies of biomarkers associated with migraine. The cohort consists of 3,893 individuals, including 1,822 complete twin pairs, 1,173 individuals with migraine and 280 migraine discordant twin pairs. The comparison between participants and non-responders revealed significant differences in both the general characteristics and the socioeconomic variables. Participants were more likely to be women, and for both genders of greater age, with a higher level of education and a higher level of income compared to non-responders. Sociodemographic differences in participation should be considered to avoid biased estimates in future studies based on the cohort.
